# Genomics and bioinformatics resources for translational science in Rosaceae

**DOI:** 10.1007/s11816-013-0282-3

**Published:** 2013-05-21

**Authors:** Sook Jung, Dorrie Main

**Affiliations:** Department of Horticulture, Washington State University, Pullman, WA 99164 USA

**Keywords:** Rosaceae, Bioinformatics, Database, Genomics, Genetics, Breeding

## Abstract

Recent technological advances in biology promise unprecedented opportunities for rapid and sustainable advancement of crop quality. Following this trend, the Rosaceae research community continues to generate large amounts of genomic, genetic and breeding data. These include annotated whole genome sequences, transcriptome and expression data, proteomic and metabolomic data, genotypic and phenotypic data, and genetic and physical maps. Analysis, storage, integration and dissemination of these data using bioinformatics tools and databases are essential to provide utility of the data for basic, translational and applied research. This review discusses the currently available genomics and bioinformatics resources for the Rosaceae family.

## Introduction

Rosaceae, comprised of over 100 genera and 3,000 species, contains a variety of crop species that are both biologically and economically important. Fruit-producing crops include apple (*Malus*), pear (*Pyrus*), raspberries/blackberries (*Rubus*), strawberries (*Fragaria*), and stone fruits (*Prunus*), such as peach/nectarine, apricot, plum, cherry and almond. Rosaceae also contains a wide variety of ornamental plants including roses, flowering cherry, crabapple, quince and pear. Crop improvement has traditionally been performed by incorporating desired traits from wild relatives through conventional breeding. Recent advances in high-throughput technology have revolutionized biology and provide unprecedented opportunities for rapid advancement of crop improvement through marker or genomics-assisted breeding.

The first spike in data generation occurred in the early 1990s when large-scale sequencing became available through the use of Sanger technology for EST and BAC sequencing (Adams et al. 1991; Shizuya et al. 1992). In the last few years, the advent of next generation technologies, such as 454 and Illumina, have significantly enhanced the ability to generate large-scale transcriptome and genome sequence at a fraction of the cost of Sanger sequencing. Similar advances in DNA array, nuclear magnetic resonance (NMR), Fourier transform infrared spectroscopy, Fourier transform ion cyclotron resonance mass spectroscopy, high performance liquid chromatography and mass spectrometry have generated large-scale gene expression, proteome and metabolome data for many species. Other data types include molecular marker data along with genetic mapping data and/or large-scale genotyping data of various varieties. More recently, high-throughput phenotypic and genotypic data are also being generated to study the interaction between genotype, phenotype and environment as well as for breeding purposes. All of these large-scale data require proper analysis, storage, integration and dissemination to enhance our understanding of biology and to be utilized in further research. Bioinformatics tools and methodologies, therefore, have become an essential and integral part of the new era of “information-driven” biological research.

Large-scale genome and transcriptome data were initially accumulated for model species, but are now available for a wide range of species. Crop plants with sequenced genomes include rice (*Oryza sativa*) (International Rice Genome Sequencing Project 2005), grapevine (*Vitis vinifera*) (Jaillon et al. 2007), sorghum (*Sorghum bicolor*) (Paterson et al. 2009), cucumber (*Cucumis sativus*) (Huang et al. 2009), maize (*Zea mays*) (Schnable et al. 2009), soybean (*Glycine max*) (Schmutz et al. 2010), cotton (Wang et al. 2012), sweet orange (Xu et al. 2012) and five species in Rosaceae: peach (*Prunus persica*), apple (*Malus domestica*) (Velasco et al. 2010), strawberry (*Fragaria vesca*) (Shulaev et al. 2011), pear (*Pyrus bretschneideri*) (Wu et al. 2013) and *Prunus mume* (Zhang et al. 2012), with red raspberry, black raspberry, apricot and plum, among others, currently being sequenced. In addition to the annotated whole genome sequences, a wealth of other genomic and genetic data is available for Rosaceae (Shulaev et al. 2008). These include BAC libraries, peach and apple physical maps, ESTs, numerous genetic maps in various species of Rosaceae and molecular markers that have been used for mapping and genotyping. Large-scale genotypic and phenotypic data are also being generated from various projects including HIDRAS (Gianfranceschi and Soglio 2004), ISAFRUIT (Audergon et al. 2009), GENBERRY (Diamanti et al. 2012) RosBREED (Iezzoni et al. 2010) and FruitBreedomics (http://www.fruitbreedomics.com). Currently, there are only limited proteomic and metabolomic data available for Rosaceae.

**Fig. 1:**
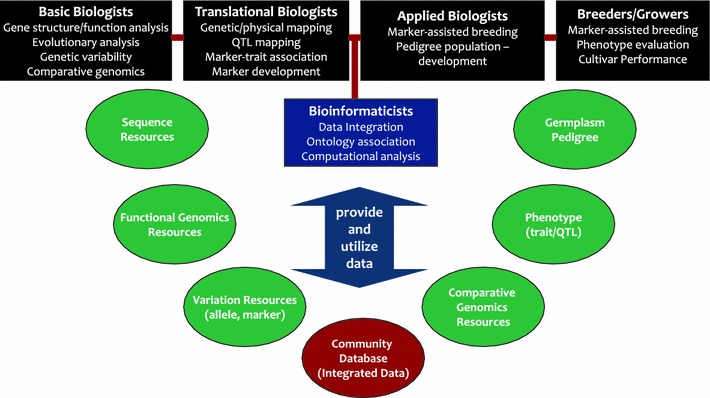
Genomics and bioinformatics resources that can be utilized by Rosaceae researchers across disciplines

In this review, we discuss genomics and bioinformatics resources for the Rosaceae family with the corresponding database resources that can be utilized for Rosaceae researchers across disciplines (Fig. 1; Table 1). Resources available in other model species are also discussed, since these present a valuable tool in conducting and future planning of research in Rosaceae.

**Table 1:** Database resources for the Rosaceae family

Name	URL	Reference
GenBank	http://www.ncbi.nlm.nih.gov/genbank/	Benson et al. (2013)
European Nucleotide Archive (ENA)	http://www.ebi.ac.uk/ena/	Cochrane et al. (2013)
DNA Data Bank of Japan (DDBJ)	http://www.ddbj.nig.ac.jp/	Kaminuma et al. (2011)
Phytozome	http://www.phytozome.org/	
CoGe (The Place to Compare Genomes)	http://synteny.cnr.berkeley.edu/CoGe/	Lyons and Freeling (2008)
The plant genome duplication database (PGDD)	http://chibba.agtec.uga.edu/duplication/	Lee et al. (2013)
Plaza	http://bioinformatics.psb.ugent.be/plaza/	Van Bel et al. (2012)
GreenPhylD	http://greenphyl.cirad.fr/v2/cgi-bin/index.cgi	Rouard et al. (2011)
SALAD database	http://salad.dna.affrc.go.jp/salad/en/	Mihara et al. (2010)
NCBI’s Gene Expression Omnibus (GEO)	http://www.ncbi.nlm.nih.gov/geo/	Wheeler et al. (2007)
ESTree DB	http://www.itb.cnr.it/estree/	Lazzari et al. (2008)
UniProt	http://www.uniprot.org/	The UniProt Consortium (2012)
The Worldwide Protein Data Bank (wwPDB)	http://www.wwpdb.org/	Berman et al. (2007)
CATH database	http://www.cathdb.info/	Cuff et al. (2011)
SUPERFAMILY	http://supfam.cs.bris.ac.uk/SUPERFAMILY/	Wilson et al. (2009)
ARAMEMNON	http://aramemnon.uni-koeln.de/	Schwacke et al. (2003)
The Arabidopsis Interactions Viewer	http://bar.utoronto.ca/interactions/cgi-bin/arabidopsis_interactions_viewer.cgi	Geisler-Lee et al. (2007)
The Biomolecular Interaction Network Database (BIND)	http://download.baderlab.org/BINDTranslation/	Isserlin et al. (2011)
The Arabidopsis Subcellular Database (SUBA)	http://suba.plantenergy.uwa.edu.au/	Heazlewood et al. (2007)
The Plant Protein Phosphorylation DataBase (P3DB)	http://p3db.org/	Yao et al. (2012)
AtMetExpress	http://prime.psc.riken.jp/lcms/AtMetExpress/	Matsuda et al. (2010)
Golm Metabolome Database (GMD)	http://gmd.mpimp-golm.mpg.de/	Hummel et al. (2010)
Genome Database for Rosaceae (GDR)	http://www.rosaceae.org/	Jung et al. (2008)

## Sequence resources

The availability of extensive sequence data forms an essential genomics resource in designing various research platforms to understand the biology of crops and to apply the knowledge in their improvements. Three primary sequence databases, to one of which researchers seeking peer-reviewed publication of new genomic data are required to submit their genome or gene sequences, are GenBank (Benson et al. 2013), European Nucleotide Archive (Cochrane et al. 2013) and the DNA Data Bank of Japan (Kaminuma et al. 2011). These databases synchronize daily, and, consequently, are generally the most up-to-date source of genomic data for any species. A caveat to this rule is where genome sequences are released ahead of publication through other portals, as in the case of the peach, sweet orange, mandarin and cotton genomes, for example, which were released through Phytozome (http://www.phytozome) ahead of publication. GenBank is built and distributed by the National Center for Biotechnology (NCBI). They collect sequence data by submission from authors and bulk submission from high-throughput sequencing centers. The Entrez search site on the homepage of NCBI (http://www.ncbi.nih.gov) allows researchers to search all the different data that it houses. Searching for information on *Prunus* species, by typing Prunus[ORGN] in the search box, returns a results page categorized by the different databases it houses. As of February 1, 2013, this search returned 131 genes, 110,815 ESTs, 7,481 unigenes, 4,931 proteins, 48,261 genome survey sequences, 46,915 nucleotide sequences, 14,705 SNPs and 89 large-scale datasets in the short read archives (SRA). For *Malus*, 61 genes, 336,190 ESTs, 22,493 unigenes, 3,523 proteins, 76 genome survey sequences, 6,410 nucleotide sequences, 7,907 SNPs and 11 large-scale datasets in the SRA. For *Fragaria*, 609 genes, 58,795 ESTs, 3,055 proteins, 17 genome survey sequences, 3,212 nucleotide sequences and 18 large-scale datasets in the SRA. Users can also download the whole genome data from their FTP site and BLAST against the whole genome. NCBI, is therefore, a good source of sequence data for Rosaceae, although access to the whole genome data is somewhat limited, and some of the larger datasets such as the SRA are not generally very useful for biologists without access to bioinformatics expertise to help with analysis of data. Researchers can keep up to date on new data submitted to NCBI for their species of interest by creating a My NCBI account which saves search history and filters data, among other features. For more information on the data and tools available at NCBI (GenBank), DDJB or EMBL (ENA), readers should refer to the excellent online tutorials at these sites and the yearly update published in the Nucleic Acids Research Database Addition published every January.

Recently, several genomes of Rosaceae have been sequenced and some of these are available from the Genome Database for Rosaceae (GDR, http://www.rosaceae.org/peach/genome, Jung et al. 2008) and Phytozome (http://www.phytozome.org/peach.php). The peach genome v1.0 is a high quality plant genome. It currently consists of eight pseudomolecules (scaffolds) representing the eight chromosomes of peach, and are numbered according to their corresponding linkage groups. The genome sequencing of the double haploid cultivar ‘Lovell’ consisted of approximately 7.7-fold whole genome shotgun sequencing employing the highly accurate Sanger methodology, and was assembled using Arachne (Batzoglou et al. 2002). The assembled peach scaffolds cover nearly 99 % of the peach genome, with over 92 % having confirmed orientation. To further validate the quality of the assembly, 74,757 *Prunus* ESTs were queried against the genome at 90 % identity and 85 % coverage, and it was found that only ~2 % were missing. While gene prediction and annotation is an ongoing process, current estimates indicate that peach has 28,689 transcripts and 27,852 genes. The whole genome sequence of apple, *Malus* × *domestica*, was reported by sequencing and assembly of the ‘Golden Delicious’ apple genome followed the whole genome shotgun approach (Velasco et al. 2010). Of the 16.9-fold genome coverage, 26 % was provided by Sanger dye primer sequencing of paired reads, and the remaining 74 % was from 454 sequencing by synthesis of paired and unpaired reads. The sequence data showed that a relatively recent (>50 million years ago) genome-wide duplication event occurred in Pyreae lineage. The domesticated apple genotypes are all highly heterozygous and the assembly produced overlapping contigs. Of 122,146 contigs, 103,076 were assembled into 1,629 metacontigs. Anchoring of metacontigs (598.3 Mb, or 71.2 % of genome) was based on the high-quality genetic map with 1,643 markers. In total, 17 linkage groups, or chromosomes, were reconstructed. The total number of genes predicted for the apple genome is 57,386, including some genes that may be present only in one of the two chromosomes of a pair. Efforts are on-going in the apple community to improve the assembly and annotation of the apple genome. A diploid strawberry, *Fragaria vesca*, has also been sequenced as a reference genome for the genus that contains the cultivated tetraploid strawberry, *Fragaria* × *ananassa* (Shulaev et al. 2011). The genome was sequenced to 39× coverage using second-generation technology, assembled de novo and then anchored to the genetic linkage map into seven pseudochromosomes. A total of 34,809 genes were predicted, with most being supported by transcript evidence. Newer version v1.1, which contains an updated pseudomolecule assembly of the original v1.0 is also available. Recently, whole genome sequencing of pear (*Pyrus bretschneideri*) and *Prunus mume* have also been reported. The pear genome was sequenced to 194× coverage using a combination of BAC-by-BAC and next generation sequencing (Wu et al. 2013). A 512.0 Mb sequence covered 97.1 % of the estimated genome size of this highly heterozygous species and 75.5 % of the sequence has been anchored to all 17 chromosomes by high density genetic maps comprising of 2,005 SNP markers. A total of 42,812 genes have been predicted with about 28.5 % encoding multiple isoforms. For *Prunus mume*, approximately 84.6 % (237 Mb) of its genome was assembled by combining 101× next-generation sequencing and optical mapping data (Zhang et al. 2012), while 83.9 % of scaffolds have been anchored to the eight chromosomes with genetic map constructed by restriction-site-associated DNA sequencing.

The whole genome sequence and annotation data of three Rosaceae species, peach, strawberry and apple, are available from GDR and Phytozome. Details on the resources and tools for the genomes in GDR are provided in the “Community database resources” section of this review. Phytozome is a joint project of the Department of Energy’s Joint Genome Institute (JGI) and the Center for Integrative Genomics to facilitate comparative genomic studies amongst green plants. Families of orthologous and paralogous genes that represent the modern descendents of ancestral gene sets are constructed at key phylogenetic nodes. These families allow easy access to clade specific orthology/paralogy relationships as well as clade specific genes and gene expansions. As of release v9.0, Phytozome provides access to 31 sequenced and annotated green plant genomes which have been clustered into gene families at ten evolutionarily significant nodes. Where possible, each gene has been annotated with PFAM (Finn et al. 2010), KOG (Tatusov et al. 2003), KEGG (Aoki and Kanehisa 2005), and PANTHER (Mi et al. 2010) assignments, and publicly available annotations from RefSeq (Pruitt et al. 2007), UniProt (The UniProt Consortium 2012) and TAIR (Lamesch et al. 2012). Similar to the GDR genome sites, users can view the genome though the genome browser GBrowse, which provides access to transcripts, alternative transcripts, mapped ESTs, aligned plant peptides and genetic markers. Users can also search their sequences against the peach genome sequence, transcripts and proteome though BLAST and BLAT servers, and download all the genome annotation through a bulk data site. The Istituto di Genomica ApplicataIGA peach site (IGA, http://services.appliedgenomics.org/projects/drupomics/intro/) also contains a peach GBrowse and BLAST tools. The IGA peach GBrowse provides access to the same tracks as phytozome but in addition they have RNA-seq Illumina profiles from tissues sequenced from cotyledon and embryo, fruit, leaf and root; repetitive sequences and microsatellites; and a profile of exact genome 20-mers.

## Resources for variation analysis

High-density genome scanning is now possible through use array platforms developed using comprising high-throughput and low-cost next generation sequencing (NGS) to identify single nucleotide polymorphisms (SNPs). The International RosBREED SNP Consortium (IRSC) *Malus* research community developed an Infinium^®^ II WGG genotyping array (IRSC array, Chagne et al. 2012) for *Malus* and *Pyrus* using data from the re-sequencing of 27 *Malus* genotypes along with data from the ‘Golden Delicious’ genome sequence. The IRSC array contains a total of 7,867 *Malus* SNPs in addition to 921 *Pyrus* SNPs. A GoldenGate-based assay platform (Khan et al. 2012) is also available for *Malus* containing 12,299 SNPs identified from EST data from 14 genotypes. Of these, 1,411 SNPs were validated using four apple genotypes. The IRSC also developed Infinium^®^ II WGG genotyping arrays for peach (Verde et al. 2012) and cherry (Peace et al. 2012). The cherry array used data from accessions of sweet cherry and of tart cherry, along with the peach genome sequence to develop a 6,000 SNP array, of which approximately one-third were informative for each crop (Peace et al. 2012).

The IRSC peach array comprised of 8,144 SNPs developed from re-sequencing of 53 peach genotypes along with data from the peach genome sequence (Verde et al. 2012).

## Functional genomics resources

### EST resources

ESTs are valuable resources for marker development, genome annotation, co-expression studies and comparative analyses as well as for gene discovery. Currently, GenBank contains 527,240 ESTs for Rosaceae: 336,190 from *Malus*, 110,815 from *Prunu*s, 58,795 from *Fragaria* and the rest from other genus. *Malus* has the largest collection of ESTs and the majority have been produced to aid discovery of genes involved in important agricultural traits with National Science Foundation funding (award no. 0321701; Schuyler Korban, principal investigator) and by HortResearch in New Zealand (Newcomb et al. 2006). Transcriptome analyses in *Prunus* focused on generating ESTs to identify candidate genes involved in different tissues, different stages of plant development and responding to abiotic and biotic stresses (Georgi et al. 2002; Horn et al. 2005; Vecchietti et al. 2009). Transcriptomics analyses have also been used to investigate fruit ripening and post-harvest physiology in *Prunus* (Trainotti et al. 2003; Vizoso et al. 2009). In *Fragaria*, 50,627 transcripts are from the diploid *Fragaria vesca* and 10,855 are from the cultivated tetraploid *Fragaria* × *ananassa*. Out of 143 SRA datasets for Rosaceae, 79 are from RNA sequences. The majority, 42 datasets, are from *Prunus* with other datasets from other genera: 12 from *Fragaria*, 10 from *Pyrus*, 9 from *Malus* and 6 from *Rosa*.

GDR contains all the publicly available Rosaceae ESTs and the unigene sets for the family Rosaceae and each genus. With the availability of the whole genome sequences, the ESTs from *Malus*, *Prunus* and *Fragaria* are anchored to the predicted gene transcripts from whole genome sequencing. The best matches between the ESTs and the predicted genes are available in a spreadsheet with links to the predicted genes in the GDR GBrowse. PlantGDB (http://www.plantttgdb.org) also contains EST unigenes, assembled from GenBank ESTs, from various plant species. Unigene data is available from *Prunus persica*, *Prunus armeniaca*, *Malus domestica*, *Fragaria vesca* and *Fragaria ananassa*. ESTree DB (Lazzari et al. 2008) has a collection of ESTs from *Prunus persica* and *Prunus amygdalus*. The ESTs are from 12 peach libraries and 3 almond libraries produced in nine different laboratories. The peach unigene in the sixth release of ESTree DB contains 28,391 unigenes with 7,709 contigs and 20,682 singlets, assembled from 75,404 ESTs. The ESTree mapping project is underway to map about 200 additional ESTs on the peach transcriptome.

### Microarrays

*Malus* has the largest collection of microarrays as well as ESTs which have been used to study environmental effects on tree-to-tree variability in the orchard, fruit aroma production (Schaffer et al. 2007), early development of apple fruit (Lee et al. 2007), apple fruit development from the floral bud to ripe fruit (Janssen et al. 2008), fruit development and ripening (Costa et al. 2010), fruitlet abscission (Botton et al. 2011), role of Ring finger gene family in fruit development (Li et al. 2011), fire blight susceptibility (Jensen et al. 2012) and apple fruit maturation and texture attributes (Zhu et al. 2012).

High-throughput transcriptome analysis using microarrays have been recently incorporated in various *Prunus* research projects. One of the platforms, microPEACH1.0, consists of 4,806 70-mer oligonucleotides designed from *Prunus persica* (peach) EST unigene clusters, mainly in the 3′ end terminal region. It was used to investigate transcriptome profiling of ripening nectarine (Ziliotto et al. 2008), stone formation in peach fruit (Dardick et al. 2010) and the role of Jasmonate in fruit ripening (Ziosi et al. 2008). GFNChile_Peach_0.9K_v1.0 contains 847 cDNAs from a cv. Loring ripe peach fruit (*Prunus persica*) cDNA library and used to study cold-responsive genes in peach fruit (Ogundiwin et al. 2008). Website (http://bioinfo.ibmcp.upv.es/genomics/ChillPeachDB) holding detailed information on the ChillPeach database was also created. Other investigations that used microarrays include the identification of key genes in almond adventitious shoot regeneration (Santos et al. 2009) and evaluation of expression of genes mostly engaged in fruit development between *Prunus mume* and *Prunus armeniaca* (Li et al. 2012). Recently, a 60-mer oligo-DNA microarray, constructed using *Prunus* Unigene V4 from GDR, was used to study fruit softening in peach (Tatsuki et al. 2013). Some of the results are available from NCBI’s Gene Expression Omnibus (GEO) (Wheeler et al. 2007).

In *Fragaria*, cDNA microarray have been used to study maturation and non-climacteric ripening (Aharoni et al. 2002) and the evolution of fruit flavor compounds (Aharoni et al. 2004) in the cultivated octoploid *Fragaria* × *ananassa*. An oligo-based microarray with sequences from both diploid and octoploid has also been used to compare the transcriptome of the ripe receptacle in these species (Bombarely et al. 2010).

### RNA-seq technology

The latest and most efficient tool for transcriptome analysis involves the use of high-throughput sequencing technologies to sequence cDNA. This technology is called Whole Transcriptome Shotgun Sequencing (WTSS) or RNA-seq. RNA-seq allows not only the assessment of the expression level of specific genes but also the detection of less-represented transcripts, allelic-specific expression of transcripts, post-transcriptional mutations and the expression of splice-variants. Even though it has been available for only a couple of years, this technology has been used extensively in humans, mammals and yeast and it is beginning to be used in plant species. *Arabidopsis* was the first plant species to be studied using this technology (Weber et al. 2007). Comparison of deep transcriptome sequencing with the EST database confirmed most of the annotated introns and identified thousands of novel alternatively spliced mRNA isoforms, suggesting at least 42 % of intron-containing genes of *Arabidopsis* are alternatively spliced (Filichkin et al. 2010). These RNA-seq data (Lister et al. 2008; Filichkin et al. 2010), along with proteomics data (Baerenfaller et al. 2008; Castellana et al. 2008), have been used to revise the *Arabidopsis* gene models and are incorporated in the TAIR10 release (Lamesch et al. 2012).

In *Malus*, RNA-seq experiments were performed to study genes associated with columnar phenotype, characterized by a compact growth habit, of apple trees (Krost et al. 2013). In *Prunus*, RNA-seq technology is currently being utilized in projects to study PPV (Plum Pox Virus) resistance, graft incompatibility fruit quality and flowering time in peach, apricot and plum, as summarized in Martínez-Gómez et al. (2011). The results are expected to be incorporated in GDR when the results are publicly available.

## Resources for proteomics and metabolomics

Proteomics and metabolomics refer to the large-scale study of proteins and metabolites. When transcriptomic, proteomic and metabolomic data are integrated, it can help to give us a more complete picture of a living organism in a specific condition. Due to the enormous complexity of the proteome, many different approaches are being taken to generate and catalogue proteomics data, such as structural and functional annotation of proteins, protein expression and dynamics, stress and developmental responses, post-translational protein modifications and protein interactions. UniProt (The UniProt Consortium 2012) provides a comprehensive, high-quality and freely accessible resource of protein sequence and functional information. Currently (searched in January 2013), 3,695, 2,495 and 1,982 protein entries are available for *Prunus, Malus* and *Fragaria*, respectively. Only 70, 45 and 34 entries of them are in Swiss-Prot and the rest (3,625, 2,450 and 1,948) are in TrEMBL.

The Worldwide Protein Data Bank (WwPDB) (Berman et al. 2007) is the main structural protein database. It includes RCSB (A Resource for Studying Biological Macromolecules) PDB in USA, PDBe in Europe (Velankar et al. 2011) and PDBj in Japan. Currently, PDB contains the coordinates and related information of more than 76,000 structures of proteins, nucleic acids, protein/nucleic acid complexes and other macromolecules that have been determined using X-ray crystallography, NMR and electron microscopy techniques. Only limited numbers of structures have been deposited in wwPDB. For *Prunus*, 11 structures are currently available from PDB including proteins from *Prunus persica*, *Prunus avium*, *Prunus dulcis* and *Prunus mume*. Other entries for the Rosaceae family include six from *Malus domestica* and one from *Fragaria* × *ananassa*. Other protein structure databases include CATH (Cuff et al. 2011) and SUPERFAMILIY (Wilson et al. 2009). The CATH (class, architecture, topology and homology) database provides a hierarchical classification of protein domain structures obtained from PDB. The classification class reflects the amino acid composition, architecture of the general shape of the protein domain and topology of the way in which the protein folds into this architecture. SUPERFAMILY provides the prediction of protein domains of known structure in amino acid sequences. The classification of domains is hierarchical, based on nature of the similarity (sequence, evolutionary and structural), class, fold, superfamily and family, following the structural classification of the protein (SCOP) database (Andreeva et al. 2008). SUPERFAMILY currently includes data for 2,476 distinct organisms, including 373 Eucaryotes. Data from *Prunus persica*, *Malus domestica* and *Fragaria vesca* are available from SUPERFAMILY.

Various web-based plant proteome-related databases are summarized in the database section of the proteomics subcommittee of the Multinational *Arabidopsis* Steering Committee Proteomics Subcommittee (MASCP) Web site (http://www.masc-proteomics.org/). Some of the databases are illustrated below. ARAMEMNON (Schwacke et al. 2003) is a database of plant membrane proteins with *Arabidopsis thaliana* as the reference model plant. Currently, the database holds all putative membrane proteins of five other plant species: grape (*Vitis vinifera*), poplar (*Populus trichocarpa*), rice (*Oryza sativa*), maize (*Zea mays*) and brachypodium (*Brachypodium distachyon*). The *Arabidopsis* Interactions Viewer (Geisler-Lee et al. 2007) is an interaction database for *Arabidopsis thaliana* predicted from interacting orthologs in yeast (*Saccharomyces cerevisiae*), nematode worm (*Caenorhabditis elegans*), fruit fly (*Drosophila melanogaster*) and human (*Homo sapiens*). The database includes 70,944 predicted and 22,156 confirmed *Arabidopsis* interacting proteins. The confirmed *Arabidopsis* interacting proteins come from BIND, the Biomolecular Interaction Network Database (Isserlin et al. 2011), high-density *Arabidopsis* protein microarrays (Popescu et al. 2007, 2009) and other literature sources. The interactions in BIND were identified using several different methods, such as yeast two-hybrid screens, but also via traditional biochemical methods. All subcellular localization data in the *Arabidopsis* Interactions Viewer are from SUBA, the *Arabidopsis* Subcellular Database (Heazlewood et al. 2007). Subcellular localization data in SUBA are brought together from various sources such as studies using chimeric fluorescent fusion proteins, proteomic surveys using mass spectrometry and literature. It also contains precompiled bioinformatic predictions for protein subcellular localizations from a set of ten different prediction tools. Complex relational queries can be performed between these experimental and predicted datasets to find and collate evidence for the subcellular location of *Arabidopsis* proteins. The Plant Protein Phosphorylation DataBase (P3DB) (Yao et al. 2012) hosts protein phosphorylation data for eight species: *Arabidopsis thaliana*, *Brassica napus*, *Glycine max*, *Medicago truncatula*, *Oryza sativa*, *Solanum tuberosum*, *Zea mays* and *Nicotiana tabacum*. AtMetExpress (Matsuda et al. 2010) and the Golm Metabolome Database (Hummel et al. 2007, 2010) are metabolome databases. AtMetExpress contains data from a study of phytochemical accumulation during development of the model plant *Arabidopsis thaliana* using liquid chromatography–mass spectrometry in samples covering many growth stages and organs. The Golm Database contains data of mass spectra from biologically active metabolites quantified using gas chromatography coupled to mass spectrometry. It covers data from mammals, yeast, corynebacterium, model plants, such as crop plants and related wild species, as well as required non-sample controls.

## Comparative genomics resources

Comparative genomics is an area of study where the structure and function of genomes from different species or varieties are compared. An important goal of comparative genomics is to obtain an insight into how genomes have evolved. Detection of duplicated regions within species and syntenic regions among species plays an important role in studies of genome evolution. The comparison of genomes and their contents among species allows us to identify genes and other sequence features that are conserved among species and/or genes that are specific to certain clades. These results help us to infer what genes and other sequence features are responsible for the similarities and differences among species. Comparative genomics also allows us to transfer knowledge from well-studied species to less-studied species.

With the increasing number of species with whole genome sequences, several web-based databases are available to compare whole genome data. A few contain the whole genome sequences of one or more species of the Rosaceae family: *Prunus persica*, *Malus domestica* and *Fragaria vesca*.

CoGe (The Place to Compare Genomes) (Lyons and Freeling 2008), contains whole genome data of 15,037 species across all domains of life, including *Prunus persica*, *Fragaria vesca* and *Malus domestica*. CoGe contains a series of web tools where users can select species of interest to view the genome data and/or perform some comparative analysis. The tools include OrganismView for searching organisms, CoGeBlast for blasting sequences against the genomes of interest, FeatView for searching genomic features by name, SynMap for whole genome syntenic dotplot analyses, SynFind for identifying syntenic regions across multiple genomes and GEvo for high-resolution sequence analysis of genomic regions. SynMap is the tool for comparative genomics which uses DAGChainer (Haas et al. 2004) as the underlying software for synteny detection.

The plant genome duplication database (PGDD) (Lee et al. 2013) contains 26 plant genomes including *Prunus persica*, *Malus domestica* and *Fragraria vesca*. PGDD contains similar tools to those in CoGE, where users can explore plant genes in terms of intra-genome or cross-genome syntenic relationships. Users can choose two species to view syntenic blocks in Dot Plot, can select a locus to view the regions that contain the locus along with the syntenic regions in multiple species, and can use BLAST to map the sequence of interest to selected genomes. PGDD uses the MCscan package (Tang et al. 2008), which uses DAGchainer to predict pairwise segments, as the underlying software for synteny detection.

Plaza (Van Bel et al. 2012) is another platform where users can access plant genome data to perform comparative analyses. Plaza contains 25 species including *Malus domestica* and *Fragraria vesca*. Data annotation includes primary gene annotation, gene family, orthologous genes, and functional annotation such as GO terms, InterPro Domains, and Reactome (pathway) data. In the Plaza Synteny Plot tool, users can start from a gene or a gene family to view the gene organization of all homologs of a gene family in selected species. In the Skyline plot, users can enter a gene locus to view the collinear regions that exist within a set of selected species. In WGDotplot, users can select two species to view syntenic blocks or select the same species twice to view all duplicated blocks within species. The collinear regions within species can also be viewed with Circle Plot. If available, the age of the collinear blocks, determined using Ks, is reported using a color code. From the Skyline plot and WGDotplot, users can access Multiplicon View to see the aligned gene strings of a set of homologous segments. The collinear regions are detected by i-ADHoRe (Simillion et al. 2008). In addition to those described above, Plaza provides other tools for genome evolution and collinearity research. The WGMapping tool allows users to choose all genes or selection of genes to display their location on the chromosomes along with the gene type. The functional clustering visualization tool provides an overview of the location and content of each functional cluster, detected using C-Hunter (Yi et al. 2007), on a chromosome-wide scale. Genomic sequences and genomic features can be viewed using Genome Browser. Tools such as Similarity heatmap, orthologous gene tool, Tree explorer and Gene family finder allows users to further explore gene family evolution.

The GreenPhylDB (Rouard et al. 2011) and SALAD databases (Mihara et al. 2010) are related databases. The GreenPhylDB contains data from *Malus domestica*, but the SALAD database does not yet contain any Rosaceae genome data. The difference between these and the comparative genome databases mentioned above is that GreenPhylDB and SALAD do not contain data or tools for synteny analysis, but focus on gene family, phylogeny and ortholog/paralog analyses. GreenPhylDB is a web resource for plant comparative and functional genomics. GreenPhylDB v3.0 contains 22 full genomes from the major phylum of plant evolution. The data include various lists of gene families, such as plant-, phylum- and species-specific lists, and tools to facilitate the comparisons. The SALAD database is a web-based resource for genome-wide comparative analysis of annotated protein sequences in plants. In SALAD, users can search for genes and view phylogenetic trees constructed from sequence alignment for a selected single motif or multiple motifs. Another functionality called sequence logo provides users with graphical representation of the sequence conservation of amino acids made from alignment of the conserved motif in each node. Users can also compare the sequence logos among members of distinct nodes to evaluate conservation and diversity of amino acid at any sites in the conserved motif.

GDR (Jung et al. 2008) uses GBrowse_Syn (McKay et al. 2010), a synteny browser, to show the orthologous regions among the three sequenced genomes of Rosaceae (Jung et al. 2012), as detected by the Mercator program (Dewey 2007). GBrowse_Syn is hyperlinked to GBrowse so that users can access various genome annotation data including markers from the conserved syntenic regions shown in GBrowse_Syn. Comparative genomics data made available in GDR thus allow users to explore other data, such as genomic features, anchored trait loci and genetic markers, in the orthologous regions.

## Community database resources

Plant community databases provide access to all or most of the datasets for individual or closely related species. Such databases include The *Arabidopsis* Information Resource (TAIR, Lamesch et al. 2012), the Genome Database for Rosaceae (GDR, Jung et al. 2008), the Solanaceae Genomics Network (SGN, Bombarely et al. 2011), Gramene (Youens-Clark et al. 2011), TreeGenes (Wegrzyn et al. 2008) and MaizeGDB (Schaeffer et al. 2011). Community databases generally store comprehensively integrated data such as annotated sequences of genomes and transcriptomes, genetic data, and molecular and phenotypic diversity data. As a result of data integration, the value of individual types of data increases exponentially, providing essential resources to accelerate the molecular understanding of phenotypic traits and the use of DNA information in crop improvement.

Initiated in 2002, GDR is the sole community database for the Rosaceae family, which includes economically important genera such as *Prunus*, *Fragaria*, *Malus*, *Pyrus*, *Rosa* and *Rubus*. The Rosaceae research community has generated data for the annotated genome sequences, physical maps, a large collection of ESTs, transcriptome map, numerous genetic maps, genetically mapped traits (MTL and QTL), genotypic diversity data, publicly available breeding data with both phenotypic and genotypic data, and cultivar evaluation data for growers. The integration and standardization of the data is crucial for the data to be utilized by different types of users including genomicists, geneticists, molecular biologists, evolutionary biologists, bioinformaticists, breeders and growers. The purpose of GDR is to collect, curate, analyze and integrate the data and provide efficient interfaces for user access to allow these numerous and complex data to be efficiently utilized. In the sections below, we describe the data and web interface, analysis tools and community tools available from GDR, together with work in progress and future plans. GDR tutorials are available at http://www.rosaceae.org/tutorials.

### Data and web interface

#### Annotated whole genome sequence

The whole genome sequences of various versions of the three species, *Prunus persica* genome v1.0, *Malus domestica* genome v1.0 and v1.0p and *Fragaria vesca* genome v1.0 and v1.1, are available in GDR. *Malus domestica* genome v1.0 is represented as metacontigs, composed of assembled overlapping contigs that have been produced by the assembly of heterozygous genotypes, which have been anchored to the chromosomes. The v1.0p is the primary pseudo-haplogype assembly, which is composed of chromosome-anchored contigs that are non-overlapping. Users can access all the annotated genome data from each genome page. Through the graphic interface GBrowse (Donlin 2007; Stein et al. 2002), users can view various genomic features aligned to the genome, such as gene models, repeats, and SNPs, as well as alignments of ESTs, repeats, genetic markers and genes from other plant model species. Each feature has hyperlinks that lead to a page with sequences and other information, with further hyperlinks to external databases where applicable. The genetic marker feature in GBrowse is linked to the marker page in GDR where all the detailed marker information is available, such as primers, mapped positions, references, and link to CMap, the comparative map viewer in GDR. The mapped ESTs are also linked to the EST page in GDR, where all the detailed EST information is available. The genome pages in GDR contains various downloadable files, including the fasta files of predicted peach gene transcripts, CDS (coding sequences), and predicted gene peptides. Excel files of gene transcripts with homologs to *Arabidopsis* genes, Swiss-Prot entries and TrEMBL entries are also available with hyperlinks to external databases. Other Excel downladable files include various Rosaceae ESTs and genetic markers that map to the whole genome sequences and SNPs with hyperlinks to GBrowse in GDR. The orthologous regions among three species, detected by the Mercator program (Dewey 2007), are displayed using GBrowse_Syn (McKay et al. 2010). The whole genome data are also available to search by various categories, such as by name, interpro protein domain name or KEGG pathway terms, so that users can directly access the genes by querying. The predicted genes from the whole genome sequences have also been utilized in the construction of PlantCyc databases. Currently, three PlantCyc databases, peachCyc, appleCyc and FragariaCyc, are available in GDR for users to explore the pathway data.

#### Annotated EST unigene data

GDR contains all the publicly available Rosaceae ESTs, downloaded from the dbEST at NCBI (Gibney and Baxevanis 2011). Routine processing in GDR occurs in three stages: sequence filtering and trimming to obtain high-quality sequences, assembly into contigs to reduce the inherent redundancy and building unigene sets from the combined contigs and singlets, and sequence annotation. A unigene is available for *Prunus*, as well as *Malus*, *Fragaria*, *Rosa* and *Pyrus*. The assembled contigs and singlets for the four genera were assembled together to generate a putative unigene set for the entire Rosaceae ESTs. Other annotation includes putative function and Gene Ontology (The Gene Ontology Consortium 2013) association to contigs and ESTs by homology with SwissProt, TREMBL and InterPRO proteins (Hunter et al. 2012). Plant Structure Ontology (Ilic et al. 2007) is also utilized to annotate the ESTs with the tissue from which the ESTs are generated.

The unigene page is a good starting point for an overview of the various annotated data for the unigenes. It displays the overall results of the project with a sidebar containing links to the library information, putative homology, KEGG analysis, microsatellite analysis and downloads. A link to the gene ontology (GO) classification is also available for the genera assemblies as well as the Rosaceae family assembly. Downloadable data includes batch sequence in fasta format, homology results file in Excel format with links into GDR and external databases and SSR/ORF/primers results in Excel format.

The EST search site is for those users who are interested in a subset of ESTs. They can choose to search ESTs of the entire Rosaceae or the genus of interest by selecting the appropriate tabs. Users can also search either ESTs or contigs. In each search page, ESTs or contigs can be searched by their name(s), assembly results, sequence features such as SSR or SNP, taxonomy, tissue type and putative function including match description, match organism and GO term. Users can also perform a batch search by uploading a file with EST names. Previous unigene versions are also available for search to help those who have been using an older version in their research. The results can be downloaded in fasta format or as a tab-delimited file with SWISS-PROT homology results containing hyperlinks back into the data for each sequence retrieved. Instead of displaying all the details on one page, the EST details page initially displays the clone information and the sequence with a sidebar containing links to library details, unigene information, sequence homology, SSR/ORF information, map position and anchored BACs when applicable. A unigene information page provides the contig name and hyperlink for both the genus and family unigenes. The contig page gives similar annotation data for the contig with additional links to the SNP results and the comprising ESTs. For the ESTs anchored to peach BACs and/or to Rosaceae genetic maps, the EST detail page provides a link to view the ESTs’ map positions using the GDR Map Viewer or CMap.

#### Genetic map

GDR currently contains data for 54 genetic maps for Rosaceae species. GDR uses CMap, the web-based comparative map tool, to allow users to compare maps from different cultivars and species. The comparative mapping facilitates the data transfer from well-studied species to less-studied ones. For example, the GDR map collection includes the TxE map (Dirlewanger et al. 2004; Howad et al. 2005), which is recognized as the reference map for *Prunus.* The TxE map, constructed from an almond × peach F2 population, contains 826 markers with a total distance of 524 cM. The TxE map contain many markers that are used in the construction of maps of other *Prunus* species such as peach, apricot, sour cherry, plum × almond–peach hybrid and almond × peach, but also other Rosaceae species such as apple and pear. The essential collinearity of the anchored markers in the *Prunus* maps and the presence of large collinear blocks among different genera in Rosaceae, such as *Prunus* and *Malus* (Dirlewanger et al. 2004), enable comparative mapping, an invaluable tool for cross-utilization of data in Rosaceae. In addition to the directly-mapped genetic markers, the TxE map in GDR-CMap displays peach transcriptome map data, major trait loci affecting agronomic characters found in various *Prunus* species (Dirlewanger et al. 2004), pathogen resistance loci (Lalli et al. 2005) and Rosaceae Conserved Orthologous Set (RosCOS) (Cabrera et al. 2009). CMap also contains the apple integrated map which was developed for anchoring metacontigs from whole genome sequencing. The integrated map were derived from six F1 populations totaling 720 individuals. The FV × FB diploid *Fragaria* reference map (Sargent et al. 2011), which played important role in the scaffold ordering of the *F. vesca* genome sequence and rose integrated consensus map, built based on the information of four diploid populations and more than 1,000 initial markers (Spiller et al. 2011) are other important resources for the community. GDR-CMap serves as an integrative tool in the utilization of the data anchored to the maps in Rosaceae. The anchored features, such as marker and ESTs, in the map are also linked to the corresponding GDR sites so that all the relevant information for the features can be viewed. Markers that are anchored to various genomes have hyperlinks to GBrowse. Another important resource in GDR is the peach physical map data. The peach physical map (Zhebentyayeva et al. 2008) is constructed from two peach BAC libraries (Georgi et al. 2002) and the physical length of the map is estimated to be 303 Mb, which is 104.5 % of the peach genome. GDR uses two tools available from the WebAGCoL Package (Pampanwar et al. 2005) to display the current peach physical map.

#### Genetic markers and traits

To provide more details of the genetic markers and traits that have been used in genetic map development or genetic diversity studies, GDR contains an extensively annotated molecular marker database. Currently, over 44,439 extensively annotated markers, including SNPs from IRSC peach array v1 (Verde et al. 2012) and candidate SNPs, are available from GDR search engine. The marker annotation includes marker aliases, source cultivar, source description, primer sequences, PCR conditions, references, map position, associated ESTs and associated BACs. While annotation of trait data is at an initial stage, the traits are annotated in GDR with aliases, published symbol, curated trait category, taxon, trait description, screening method, map position and references.

The marker search site allows both a simple search by name and an advanced search with various search categories. The search category includes marker type, the species from which the marker is developed, the species to which the marker is mapped, map position, markers with associated BAC clones and markers with associated ESTs. Users can also upload a file of names to get the detailed data. In the trait search site, users can search trait by name, symbol, taxon or curated trait category. Other SNP markers included in arrays, such as IRSC apple 9K (Chagne et al. 2012), cherry 6K (Peace et al. 2012) and UC Davis peach 6K (Ahmad et al. 2011), as well as those in IRSC peach 9K, are available to download and view in GBrowse.

#### DNA polymorphism

GDR contains DNA polymorphism data from various projects on molecular diversity studies of Rosaceae species. Currently, data from nine different projects are available, three from *Prunus* diversity studies and the rest from *Malus* and *Pyrus* studies. All the current data are from projects with SSR markers. Users can query by the marker name or species to view the details of diversity projects and the genotype of the varieties used in the analyses. SNP markers from the RosBREED project (http://www.rosbreed.org) are being added.

#### Breeding data

One of the newest components of GDR is the search/browse site for breeding data. GDR contains password-protected private breeding data as well as publicly available breeding data. Private breeding data includes data from the Washington Apple Breeding Program and Pacific North West Sweet Cherry Breeding Program. Public data includes data from the federally funded RosBreed project, a program designed to establish a sustainable marker-assisted breeding infrastructure for US Rosaceae crops. The data includes the varieties and their pedigrees, phenotyping and genotyping data and experimental metadata. The current search interfaces allows users to search by datasets, variety name, trait values and pedigree. From the result page, users can view detailed results for a variety or download an Excel file with all the phenotyping/genotyping results that has been specified. Users can also select a pedigree by selecting a variety and number of ancestral and progeny generations to generate an input file for breeding software such as Pedimap and FlexQTL (Bink and van Eeuwijk 2009). A breeding decision tool called Cross Assist, which produces a list of parents to cross and the number of seedlings to screen to obtain certain number of seedlings above/within user-specified trait thresholds, is also available.

### Analysis tools

GDR web-based tools include a BLAST server, FASTA server, CAP3 Assembly server and SSR server. The FASTA/BLAST servers allow users to conduct sequence homology analyses against various sequence databases including annotated sequences in GDR. The databases include whole genome nucleotide and protein sequences of peach, strawberry and apple; ESTs of the Rosaceae or each genus from NCBI, genera-specific unigene sets, as well as a family-wide unigene; Rosaceae genomic or protein sequences from NCBI, peach, apple and cherry SNP sequences; and *Arabidopsis* protein sequences from TAIR and the ESTs, unigene sets, SSR-containing ESTs from individual cDNA libraries of peach mesocarp, almond, octoploid strawberry and diploid strawberry. Peach mesocarp ESTs that are anchored to the peach BACs are also available for sequence analysis. Batch sequences can be uploaded for analysis and the results are returned as both raw aligned output and parsed out in Excel. The output in Excel has hyperlinks to the GDR and NCBI sites. FASTA formatted library files of both the sequences with or without matches are provided to allow the user to easily conduct further batch searches in GDR and other databases. An EST assembly server using the CAP3 program is also available so that users can assemble their own EST sets. The server returns the raw output, a summary report and a fasta file containing the combined contig sequences and singlet sequences, which are also available as individual files. The contig file lists the contig number and comprising clone names in the comment line for each assembled transcript. Also available is a SSR server that allows user-defined SSRs to be identified in uploaded sequences. Users can also choose to run Primer3 along with the SSR-detection program to generate primer sets for the SSRs. The results are returned in an Excel file containing all the SSRs, primers, ORFs in sequence and product size with summary information on motif number and type.

### Community resources

GDR provides access to community-based news on various pages under the ‘community’ header bar, such as Rosaceae genomics, USRosEXEC, conferences, meetings, funding, employment, mailing lists and message boards. USRosEXEC stands for US Rosaceae Genomics, Genetics and Breeding Executive Committee, which serves as a communication and coordination focal point for the community. The USRosEXEC page provides the official documents, meeting minutes, membership and subcommittee information. Several mailing lists, in addition to the GDR mailing list, are available to serve the community with information for specific interests or purposes, and the archives can be viewed through the message board sites. All the publications in Rosaceae genomics and genetics are also available in GDR through the publication search site.

### Future directions

With the availability of the whole genome sequences of apple, peach and strawberry, and other Rosaceae genomes and re-sequencing data to be added, the future direction of GDR will include further integrating the annotated whole genome data with other genomics, genetics and breeding data to accelerate the usage of DNA information in crop improvement as well as to improve our knowledge on various aspects of Rosaceae biology. The genetic markers will also be queried by the anchored genome position and the neighboring trait locus. The effort toward collecting and curating trait data, including MTL and QTL, will also be continued. When data for genetic markers, DNA polymorphism and trait data are integrated and easily searchable, users will be able to query for markers that are close to the trait of interest and polymorphic between the varieties of interest. The Genome Sequence Annotation Server (GenSAS) (Lee et al. 2011), an online annotation tool that provides a customizable automated pipeline for whole genome structural annotation, will be available for community for further curate the genome. In collaboration with RosBreed project, more breeding decision tools will be developed. Growers’ gateway that allows growers to get information about cultivars and compare the cultivar performances will also be developed.

## Conclusion

The recent addition of publicly available whole genome sequences of three Rosaceae species to the already existing wealth of genetic data has brought great opportunity for researchers to accelerate Rosaceae research. Multiple whole genome sequences in one family allow genome level comparative analysis to gain evolutionary insight as well as transfer knowledge among species. The second and third generations of high-throughput sequencing technology now allows resequencing of multiple varieties to catalogue sequence variations and quantitative gene expression analyses. The proteome and metabolome analyses are still in their infancy in Rosaceae, but the advances of tools and methodology with other model species will aid greatly in planning and adopting those analyses in Rosaceae research. Integration of different types data is critical in the interpretation and utilization of these data and hence the role of the community databases to bring together genomics, genetics, breeders and growers data will become increasingly important.

## Abbreviations

BAC: Bacterial artificial chromosome
BIND: Biomolecular interaction network database
CoGe: Place to compare genomes
EST: Expressed sequence tag
GDR: Genome database for Rosaceae
GEO: Gene expression omnibus
GO: Gene ontology
IGA: Istituto di Genomica Applicata
IPGI: International Peach Genome Initiative
JGI: Joint Genome Institute
KEGG: Kyoto Encyclopedia of Genes and Genomes
KOG: Eukaryotic Orthologous Groups
MASCP: Multinational *Arabidopsis* Steering Committee Proteomics Subcommittee
MTL: Mendelian trait loci
NCBI: National Center for Biotechnology
NMR: Nuclear magnetic resonance
ORF: Open reading frame
P3DB: Plant Protein Phosphorylation DataBase
PANTHER: Protein Analysis through Evolutionary Relationships
PGDD: Plant Genome Duplication Database
PPV: Plum pox virus
QTL: Quantitative trait loci
RCSB: Resource for Studying Biological Macromolecules
RosCOS: Rosaceae Conserved Orthologous Set
SCOP: Structural Classification of the Protein database
SGN: Solanaceae Genomics Network
SNP: Single nucleotide polymorphism
SRA: Short read archives
SSR: Simple sequence repeat
SUBA: *Arabidopsis* Subcellular Database
TAIR: *Arabidopsis* information resource
USRosEXEC: US Rosaceae Genomics, Genetics and Breeding Executive Committee
WTSS: Whole Transcriptome Shotgun Sequencing
WwPDB: Worldwide Protein Data Bank
